# Role of biofilms in antimicrobial resistance of the bacterial bovine respiratory disease complex

**DOI:** 10.3389/fvets.2024.1353551

**Published:** 2024-06-12

**Authors:** Sara Andrés-Lasheras, Rahat Zaheer, Murray Jelinski, Tim A. McAllister

**Affiliations:** ^1^Lethbridge Research and Development Centre, Agriculture and Agri-Food Canada, Lethbridge, AB, Canada; ^2^Western College of Veterinary Medicine, University of Saskatchewan, Saskatoon, SK, Canada

**Keywords:** biofilms, antimicrobial resistance, bovine respiratory disease, *Mannheimia haemolytica*, *Pasteurella multocida*, *Histophilus somni*, *Mycoplasma bovis*

## Abstract

An increase in chronic, non-responsive bovine respiratory disease (BRD) infections in North American feedlot cattle is observed each fall, a time when cattle are administered multiple antimicrobial treatments for BRD. A number of factors are responsible for BRD antimicrobial treatment failure, with formation of biofilms possibly being one. It is widely accepted that biofilms play a role in chronic infections in humans and it has been hypothesized that they are the default lifestyle of most bacteria. However, research on bacterial biofilms associated with livestock is scarce and significant knowledge gaps exist in our understanding of their role in AMR of the bacterial BRD complex. The four main bacterial species of the BRD complex, *Mannheimia haemolytica*, *Pasteurella multocida*, *Histophilus somni*, and *Mycoplasma bovis* are able to form biofilms *in vitro* and there is evidence that at least *H. somni* retains this ability *in vivo*. However, there is a need to elucidate whether their biofilm-forming ability contributes to pathogenicity and antimicrobial treatment failure of BRD. Overall, a better understanding of the possible role of BRD bacterial biofilms in clinical disease and AMR could assist in the prevention and management of respiratory infections in feedlot cattle. We review and discuss the current knowledge of BRD bacteria biofilm biology, study methodologies, and their possible relationship to AMR.

## 1 Introduction

**Figure 1:**
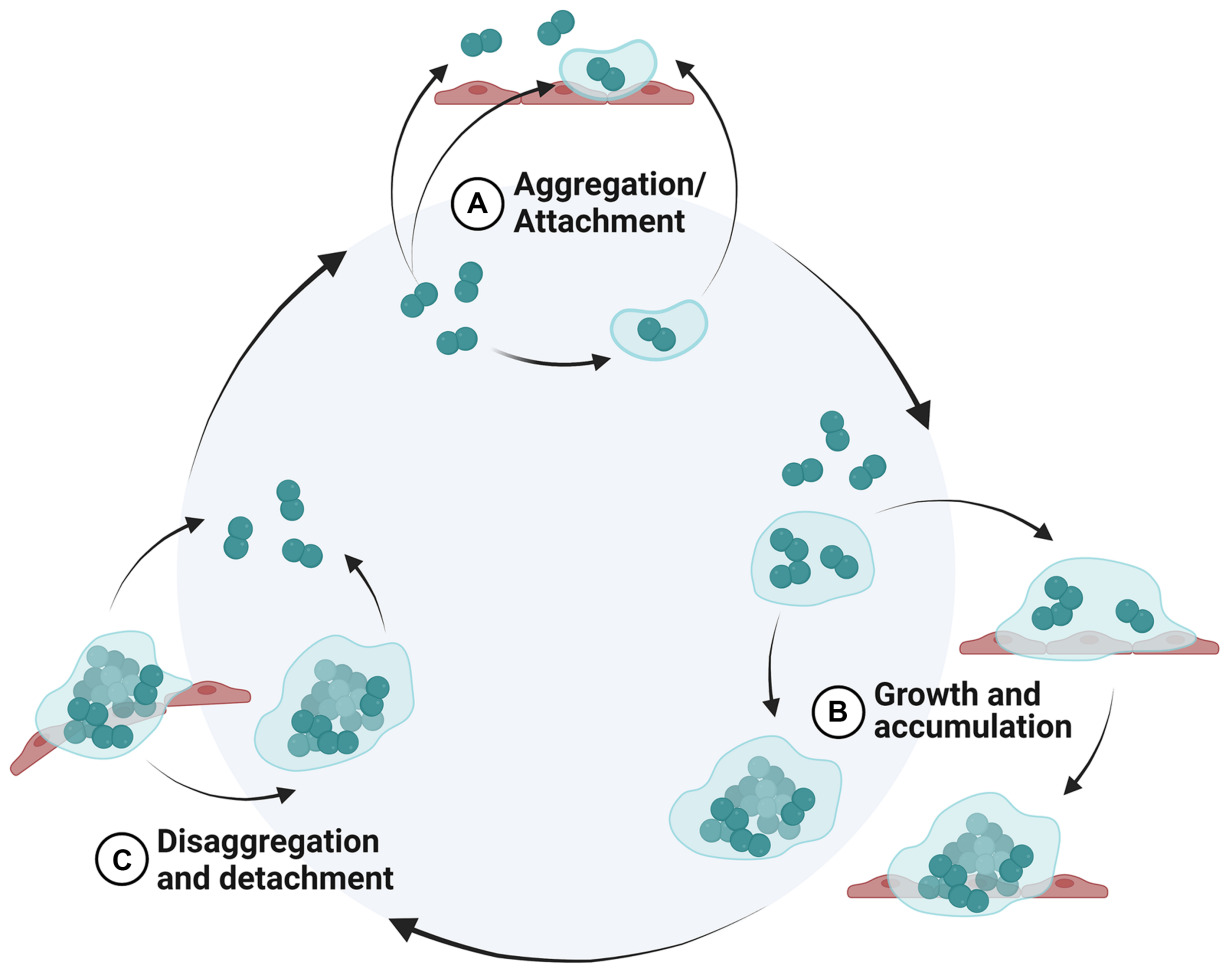
Biofilm life cycle. New proposed model of a biofilm life cycle modified from (1). Inside the light blue circle represents non-surface-attached biofilm aggregates, whereas outside the circle represents the surfaced-attached biofilm. **(A)** Bacterial cells aggregate or attach to a biotic or abiotic surface, **(B)** bacterial cell colonies increase in size by cell division (growth) and capturing surrounding cells (accumulation), **(C)** bacterial cells are sloughed from the biofilm as single cells or aggregates. *Created in BioRender*.

Bacterial biofilms are highly organized communities that can adhere to biotic or abiotic surfaces or thrive as non-surface-attached aggregates (1). They are embedded in a self-produced extracellular matrix (ECM) formed of polysaccharides, nucleic acids, and proteins also known as extracellular polymeric substances (EPS; Figure 1). Compared to free-living (planktonic) bacteria, biofilm communities are known to be more resistant to host immune responses, chemical disinfectants, and other environmental stressors such as antimicrobials (2). It was suggested that planktonic single cells may be a transitory state and that biofilms are the default lifestyle of bacteria (3), which can be 1,000 times more resistant to antimicrobials (4). It is well-established that human chronic infections caused by bacteria are often mediated by biofilms, as it is estimated they are involved in ⁓ 80% of human infections like those caused by *Pseudomonas aeruginosa*, *Staphylococcus epidermidis*, *Staphylococcus aureus*, or *Escherichia coli* (5, 6). Research on biofilms associated with livestock species is scarce. In Veterinary Medicine, biofilms can play an important role in otitis externa and endometritis in dogs (by *Staphylococcus intermedius* and *P. aeruginosa*), mastitis in dairy cattle (mainly caused by *Staphylococcus aureus*), and pneumonia in swine (*Mycoplasma hyopneumoniae*) (2). Aside from causing chronic infections, biofilms promote the survival of bacteria within the host until conditions are such that clinical disease ensues (7).

**Figure 2:**
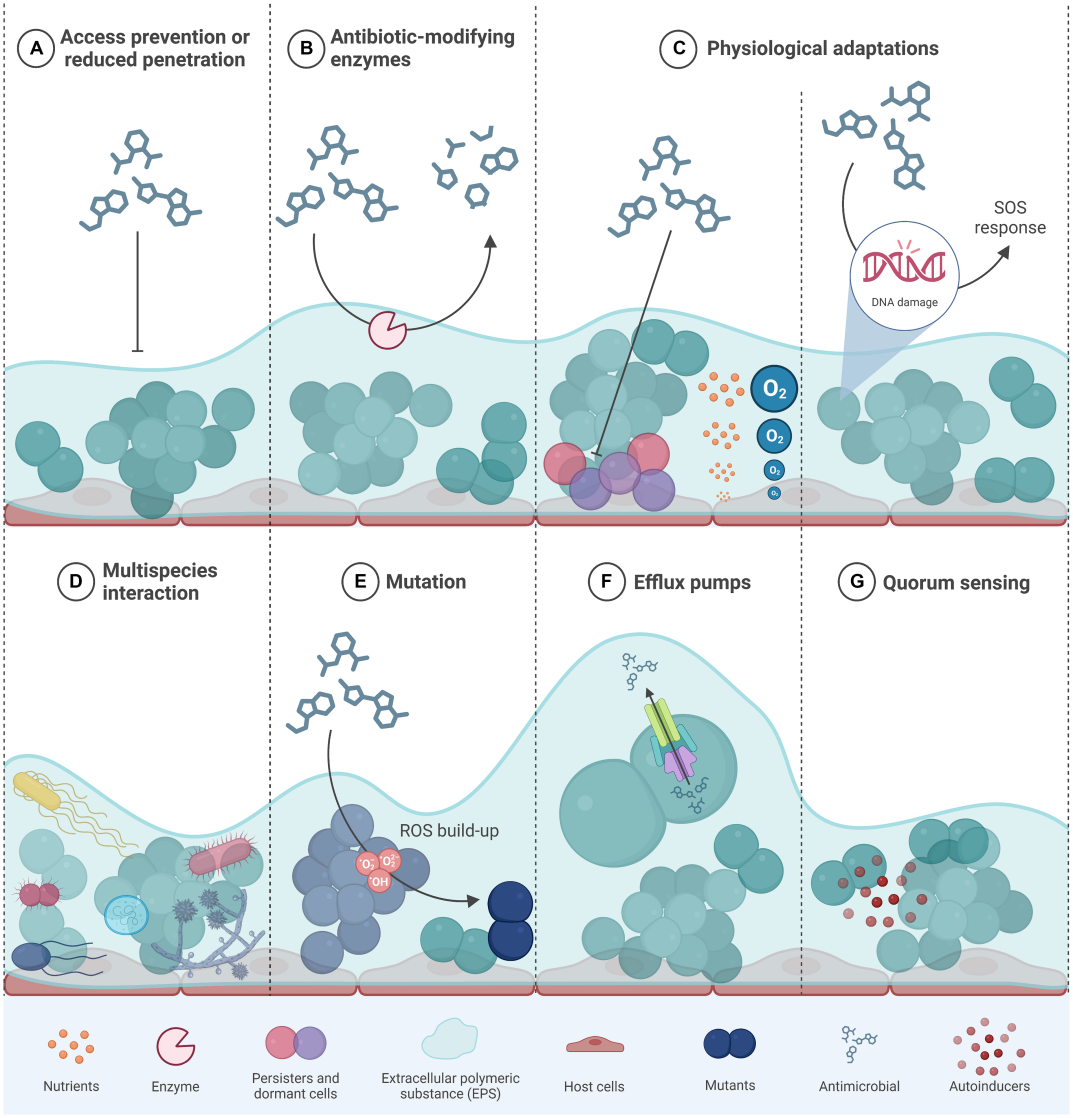
Different mechanisms that confer antibiotic resistance and/ or tolerance to bacteria within a biofilm. Modified from (4). **(A)** the components of the extracellular matrix form a physical barrier to diffusion of antimicrobials; **(B)** the presence of antibiotic-modifying enzymes secreted by some bacteria within the biofilm provides protection to both antimicrobial resistant and susceptible cells; **(C)** biofilms generate concentration gradients of nutrients or oxygen that promote the emergence of dormant cells and/or persister cells within the deeper biofilm layers; **(D)** through quorum sensing, bacterial cells from different species communicate so as to trigger physiological responses that decrease susceptibility to antimicrobials; **(E)** antimicrobials that target DNA trigger the SOS response upon DNA damage that ultimately confers AMR through other mechanisms such as mutation; **(F)** some efflux pumps are capable of transporting antimicrobials outside of the bacterial cell, thus preventing their deleterious effects; **(G)** quorum sensing bacterial cell–cell communication systems are involved in the synthesis and structure of biofilms and in cellular responses that can confer AMR; AMR, antimicrobial resistance; eDNA, extracellular DNA; ROS, reactive oxygen species. *Created in BioRender*.

As the name implies, bovine respiratory disease (BRD) refers to any respiratory disease of cattle, which frequently occurs in young beef and dairy calves. However, BRD is most commonly associated with pneumonia in beef cattle after feedlot arrival, giving rise to it being described as “shipping fever” as clinical symptoms are seen in calves following shipping from ranches and auctions to feedlots (8). Shipping fever is a multifactorial disease with a constellation of risk factors such as inclement weather, management practices, and microbial pathogens contributing to pathogenesis. Aside from viruses (bovine herpes virus 1, bovine virus diarrhea virus type 1 or type 2, bovine respiratory syncytial virus, and parainfluenza virus 3), the bacteria most commonly associated with BRD include the *Pasteurellaceae* spp. (*Mannheimia haemolytica, Pasteurella multocida*, *Histophilus somni*) and *Mycoplasma bovis* (8). Two additional bacterial species, *Bibersteinia trehalosi* and *Trueperella pyogenes*, are less frequently associated with BRD in North America (9). BRD accounts for 70–80% of feedlot morbidities and 40–50% of total feedlot mortalities, costing the North American feedlot cattle industry over 3 billion $ per year (10). Despite the advent of new antimicrobials and vaccines, and the existence of pre-conditioning programs, the burden BRD poses on the North American feedlot industry has remained largely unchanged over the last 45 years (11). The epidemiology of BRD is well-known, and hence metaphylaxis is frequently administered to calves upon feedlot arrival with the aim of mitigating the incidence and severity of BRD. *Pasteurellaceae* infections usually respond favorably if antimicrobials are administered during the early stages of BRD (12, 13). But during BRD peak season (the fall in Canada and the US), there is an increase in chronic respiratory infections, which strains feedlot resources (i.e., chronic pen capacity, personnel availability and morale) and presents an animal welfare concern and an economic burden (13). One of the challenges of BRD is its polymicrobial nature since different BRD bacteria and/ or viruses can coexist within the same calf (14, 15). Chronically infected cattle frequently have a history of receiving multiple antimicrobials, a practice that can select for antimicrobial-resistant (AMR) bacteria, which in turn could lead to a reduced therapeutic efficacy (16). Aside from AMR, there are additional factors that may contribute to BRD antimicrobial treatment failure such as viral infection, advanced state of the disease, insufficient antimicrobial concentration reached at the site of infection, antimicrobial handling/ storage under sub-optimal conditions, and possibly biofilms (Figure 2) (4, 13). To date, only *H. somni* biofilms have been confirmed to occur *in vivo* in cattle with clinical disease (17), whereas further research is required to determine if *M. haemolytica* and *P. multocida* form biofilms within the respiratory tract (18, 19). *Mycoplasma bovis* causes severe chronic damage to the lungs of calves and is responsible for chronic pneumonia and polyarthritis syndrome (CPPS) in feedlot cattle (20). *M. bovis* is capable of forming prolific *in vitro* biofilms (21) that readily form on inert surfaces and dead tissue (22). Therefore, biofilm formation, combined with antimicrobial resistance, could be a major player in the chronic nature of *M. bovis* infections. Regarding *B. trehalosi* and *T. pyogenes* biofilms, to date, no peer-reviewed literature is available related to the feedlot cattle respiratory tract.

To what extent biofilms may play a role in BRD is largely unknown (2, 17–19). A better understanding of the possible role of BRD bacterial biofilms in clinical disease could assist in the prevention and management of respiratory infections in feedlot cattle. Considering that the North American cattle industry is moving toward precision metaphylactic use of antimicrobials (AMU) in calves^1^, an understanding of the role biofilms in AMR and BRD infections is imperative.

## 2 Search strategy

In July 2023, a search building strategy was followed through the medical subject headings (MeSH) at the PubMed website (accessed on July 3rd, 2023),^1^ to systematically identify peer-reviewed articles about BRD bacteria biofilms. The following searches were carried out: (“Biofilms”[Mesh]) AND “Bovine Respiratory Disease Complex”[Majr]; (“Biofilms”[Mesh]) AND “*Pasteurella multocida*”[Mesh]; (“Biofilms”[Mesh]) AND “*Mannheimia haemolytica*”[Mesh]; (“Biofilms”[Mesh]) AND “*Haemophilus somnus*”[Majr] (*Histophilus somni*); (“Biofilms”[Mesh]) AND “*Mycoplasma bovis*”[Majr]; (“Biofilms”[Mesh]) AND “*Trueperella pyogenes*”[Mesh]; (“Biofilms”[Mesh]) AND “*Arcanobacterium pyogenes*”[Mesh] (“Biofilms”[Mesh]) AND “*Actinomyces pyogenes*”[Mesh] (“Biofilms”[Mesh]) AND “*Corynebacterium pyogenes*”[Mesh] (“Biofilms”[Mesh]) AND “*Bibersteinia trehalosi*”[Mesh].

Additionally, the following keyword terms were searched in (non-MESH) PubMed and Scopus (accessed on July 3rd 2023;^2^ only articles published in English were considered): Bovine respiratory disease biofilm; *Pasteurella multocida* biofilm; *Mannheimia haemolytica* biofilm; *Histophilus somni* biofilm; *Mycoplasma bovis* biofilm; *Trueperella pyogenes* biofilm; *Arcanobacterium pyogenes* biofilm; *Actinomyces pyogenes* biofilm; *Corynebacterium pyogenes* biofilm, and *Bibersteinia trehalosi* biofilm. Any peer-reviewed manuscripts cited in the literature identified by the aforementioned searches that addressed the biology of BRD bacterial biofilms were also included.

As a systematic review, all the articles identified by the described methodology were included. Those articles addressing BRD-related bacterial biofilms in non-bovine hosts were included as well, e.g., *P. multocida* associated with fowl cholera or respiratory infections in swine. In total, 46 BRD biofilm studies were included.

## 3 Biofilm biology in BRD bacteria

### 3.1 Mannheimia haemolytica

Morck et al. observed glycocalyx-encased microcolonies of Gram-negative bacteria with morphology compatible with *M. haemolytica* (*Pasteurella haemolytica* at the time) in lung tissue from calves with fibrinous pneumonia (16). *M. haemolytica* was isolated from the diseased lung tissue, but the bacterial species contained in microcolonies were not confirmed. Subsequently, Olson et al. (23) documented higher AMR in *M. haemolytica* biofilms compared to planktonic cells, an observation supported by a later study (24). The first scanning electron microscopy images of *M. haemolytica* biofilms were published in 2011 (25) and in 2015, Boukahil and Czuprynski demonstrated that *M. haemolytica* formed protein-rich biofilms on plastic surfaces (24). Among the proteins identified, OmpA, OmpP2 (an amyloid-like protein) and OmpH (a porin protein) were particularly prevalent (26, 27).

Boukahil et al. showed that *M. haemolytica* formed biofilms on fixed primary bovine epithelial cells and that mucin inhibited biofilm formation (28). The formation of biofilms *in vitro* was also greater for *M. haemolytica* isolates from the deep lung of pneumonic cattle as compared to those obtained from the nasopharynx of healthy cattle (29). However, *M. haemolytica* isolates used in this study were not serotyped to confirm if they were either A2 or A1, which are more likely to be associated with healthy versus diseased calves, respectively. The same study demonstrated that stress-induced molecules in cattle (norepinephrine, epinephrine, and substance P, a small peptide belonging to neurokinins) promoted biofilm dispersion. It is widely accepted that stress caused by the transportation of calves to feedlots can promote the proliferation of *M. haemolytica* serotype A1 in the upper respiratory tract, increasing the risk of it flowing to the lower respiratory tract where it can cause disease (29). Therefore, it was hypothesized that these 3 stress-related elements could promote the disruption of *M. haemolytica* biofilms and the movement of this pathogen into the lower respiratory tract.

### 3.2 Histophilus somni

*Histophilus somni* causes fibrino-suppurative bronchopneumonia and septicemia as well as polyarthritis, myocarditis, pericarditis, pleuritis, and laryngitis in feedlot cattle (17). It is capable of forming biofilms both *in vitro* and *in vivo* (17, 30–33). It is speculated that commensal and pathogenic *H. somni* strains form biofilms that differ in architecture and shape. Previous studies reported that a commensal strain formed a thin, heterogeneous, and filamentous biofilm whereas a pathogenic strain formed thick, homogenous, mound-shaped microcolonies encased in an amorphous extracellular matrix with prominent water channels (30). The EPS of *H. somni* biofilms is a branched mannose polymer with occasional terminal galactose residues whose synthesis is enhanced in the presence of increased concentrations of salt or with anaerobiosis (34). *In vivo H. somni* biofilms were detected in the cardiopulmonary tissue of cattle after experimental challenge, supporting their role in pathogenesis (17). Others have described *H. somni* biofilms on bovine myocardial and brain microvascular endothelium, suggesting they play a role in the pathogenesis of *H. somni* mediated heart and brain infections (32).

Different molecular processes that have an impact on *H. somni* biofilm formation have been described in the literature. Universal stress proteins (Usp) are ubiquitously related to stress responses and adaptation in bacteria. The UspE protein is thought to be a global regulator in *H. somni* and is associated with biofilm formation, EPS production, and lipooligosaccharide (LOS) truncation (35). A *H. somni* mutant strain 2,336::Tn*uspE* failed to produce biofilms *in vitro* and was EPS deficient when grown on glass coverslips in well plates (35). Comparative transcriptomics of mutant and wildtype strains grown under planktonic and biofilm states revealed that 181 genes were differentially expressed between the two cell forms. Quorum sensing (QS) plays an important regulatory role in biofilm formation in many bacteria. The *luxS* gene encodes an enzyme involved in the biosynthesis of AI-2, a QS small molecule thought to be a universal signaling component in bacteria. However, *luxS H. somni* mutants showed no impaired biofilm formation compared to a wildtype *H. somni* strain (35), suggesting that luxS may not play a role the formation of biofilms by *H. somni*. Small RNAs (sRNAs) primarily have post-transcriptional gene regulatory functions in bacteria; sRNAs HS9 and HS97 were shown to interact with the transcription factor sigma 54, which may influence biofilm formation and other functions in *H. somni* (23).

### 3.3 Pasteurella multocida

There are 5 serogroups of *P. multocida* based on their capsular type or capsular polysaccharide (CPS): A, B, D, E and F. Like *M. haemolytica*, *P. multocida* biofilm formation has been demonstrated *in vitro* but only indirectly *in vivo* in cattle (19, 36–40). The exopolysaccharide (ExPS; biofilm matrix) of *P. multocida* type A mainly consists of glycogen and proteins, but the protein fraction in *P. multocida* biofilms maybe less prominent than in other bacterial species (7, 40). The amount of capsule synthesis in *P. multocida* appears to be inversely related to its biofilm formation capabilities, a trait that does not seem to be limited to any specific serogroup and can be reversed through mutagenesis or *in vitro* passage (7). Capsular inhibition of biofilm formation is presumably a consequence of the blockage of proteins essential for adhesion to surfaces (7). Petruzzi et al. proposed a *P. multocida* transmission model for fowl cholera in which biofilms result in chronic infections, whereas planktonic *P. multocida* was associated with acute, symptomatic infections (7, 19). However, it is unknown if this model is applicable to *P. multocida* infections in cattle.

Several studies have identified specific genes in *P. multocida* that are inovled in cell adhesion and biofilm formation. A complete cluster of seven *tad* genes (*tadABCDEFG*; *tad* locus) related to nonspecific adherence in *Actinobacillus actinomycetemcomitans* were found to be highly conserved in a *P. multocida* isolate (41). In *P. multocida* strain HB03 (GenBank accession no. CP00332), the *tad* locus contained the 14 intact genes that this locus typically harbors, i.e., *flp1-flp2-tadV-rcpCAB-tadZABCDEFG* (42). However, the specific involvement of *tad* genes in biofilm formation in *P. multocida* requires further evaluation as they are not conserved across all strains (38, 39, 42–45). The *csrA* gene is involved in glycogen synthesis and as a result, it plays a role in biofilm formation (7). Other *P. multocida* genes with possible direct or indirect biofilm regulation roles include *xylB*, *sgbU*, *hexD, aspA, comE, qseC, and pmorf0222* (7, 43, 46–48). Of special interest is the *qseC* gene and its role in QS modulating the transcription of thousands of genes. This highlights that biofilm formation in *P. multocida* may be a complex biological process, that is likely influenced by major stress-responding molecular mechanisms (47).

### 3.4 Mycoplasma bovis

*Mycoplasma bovis* is considered one of the most pathogenic Mycoplasma species of cattle, causing a broad spectrum of diseases that includes arthritis, genital disorders, mastitis, pneumonia, keratoconjunctivitis, and otitis media (49). Due to genome erosion, mycoplasmas have lost a substantial number of genes including some essential for cell survival and those related to cell wall synthesis (degenerative evolution). Therefore, they have a limited ability to synthesize macromolecules needed for cell growth (50). As a consequence, most mycoplasmas have adopted a parasitic, facultative intracellular symbiotic life style where they depend on their host to provide fatty acids, nucleic acid precursors, lipid precursors, amino acids, and vitamins (51, 52). However, some studies have reported that *M. bovis* can survive in environmental samples for up to 8 months or after surface washing and disinfection (53–56), which suggest that it may form biofilms in these environments.

Eighty-one different isolates from 11 different mycoplasma species of veterinary interest were tested for their ability to form biofilms (21). These included *M. bovis, M. mycoides* subsp. *mycoides*, and *Mycoplasma* spp. bovine group 7. Differences in biofilm formation were observed, both across and within species. Some *M. bovis* isolates formed prolific biofilms, while others were less abundant. These findings support the hypothesis that *M. bovis* did not lose its ability to form biofilms as a result of degenerative evolution, which may speak to the importance of its role in bacterial survival. A correlation between the expression of specific variable surface proteins (Vsp; related to adherence) and biofilm capabilities have been described in *M. bovis* (21). Moreover, *M. bovis* biofilms showed more resistance to heat and desiccation, but not to antimicrobials like oxytetracycline, danofloxacin, and enrofloxacin (21). However, heat and desiccation were tested using 48-h old biofilms whereas resistance to antimicrobials was assessed using 24-h old biofilms, raising the possibility that biofilm maturity may have influenced responses.

Chen et al. used the whole-cell protein fraction in an immunoproteomics study to characterize the host response to *M. bovis* infections and identify new vaccine and diagnostic targets. Convalescent sera samples were obtained from calves experimentally infected with *M. bovis*. Six immunoreactive proteins specific to biofilms, but not to planktonic cells were detected, with most being of cytoplasmatic origin (57). The detection of biofilm antigens in convalescent sera provided indirect evidence of the involvement of biofilms in *M. bovis* infections.

### 3.5 Trueperella pyogenes and Bibersteinia trehalosi

In North America, the identification of these two bacterial species in BRD studies is limited (9), presumably due to their infrequent involvement in clinical BRD. The search strategies defined in the M&M section identified no publications related to *B. trehalosi* biofilms. Regarding *T. pyogenes*, several biofilm peer-reviewed studies were identified; however, none of them included *T. pyogenes* isolates originating from the respiratory tract of feedlot cattle.

*Trueperella pyogenes* chronic infections have been associated with biofilms in mastitic and metritic dairy cows and are thought to contribute to unresponsiveness of these conditions to antimicrobial therapy (58, 59). Even though *T. pyogenes* isolated from infections in dairy cows and forest musk deer has been shown to form biofilms *in vitro* (58, 60–62), *in vivo* biofilm formation has yet to be formally demonstrated. Only one study reported the *in vivo* formation of *T. pyogenes* biofilms in mice (60). However, biofilms were detected on hydro-gel contact lenses (i.e., surgically introduced in the murine reproductive system before *T. pyogenes* challenge) rather than on the uterine tissue. It has been reported that *P. aeruginosa* and *E. coli* may inhibit *T. pyogenes in vitro* biofilm production through the production of QS signal molecules, which may explain the progression toward dominance of these bacterial species in the abscesses of musk deer (63). Studies related to the molecular regulation of *T. pyogenes* biofilms found that the expression of the virulence factors hemolytic exotoxin pyolisin (PLO) and TatD DNases are linked to biofilm formation (58, 64). The *ploS*/*ploR* genes from the two-component regulatory system, and the *luxS* gene may also play a role in the formation of biofilms by *T. pyogenes* (65).

## 4 Polymicrobial BRD biofilms

Not many studies have addressed the polymicrobial nature of BRD infections in biofilm experiments. *Mannheimia haemolytica* and *P. multocida* inhibited biofilm formation by contact-dependent inhibition, a type of inhibition that is mediated by cell adhesion of one species to another (66). In contrast, *in vivo H. somni* biofilm studies revealed the presence of *Pasteurella multocida* in the respiratory tract of calves, suggesting the existence of polymicrobial BRD bacteria biofilms (17). Later, a possible synergistic relationship between *H. somni* and *P. multocida* in biofilm formation was described (40). In this study, it was observed that when both species were grown together *in vitro*, the average biomass and biofilm thickness, and the total carbohydrate and protein content of the biofilm were greater than in single-species biofilms. It was suggested that *H. somni* cells interact with *P. multocida* cells to enhance aggregation and the formation of polymicrobial biofilms. Furthermore, gene expression in *P. multocida* was altered in a manner that increased its persistence and ability to contribute to the formation of multi-species biofilms with *H. somni.* When two 18-month old calves were challenged with *H. somni* and sera was collected, Petruzzi et al. detected a more robust (bovine) host immune response (ELISA) to biofilms compared to planktonic cells of *H. somni* or *P. multocida*. These experiments highlighted the possible role of polymicrobial biofilms in BRD infections (39). The detection of *P. multocida* while studying *H. somni* biofilms was a serendipitous but feasible finding, as the three *Pasteurellaceae* species have common nutritional requirements (17, 40, 66). The lack of detection of *M. bovis* in these multi-species biofilms could be a reflection of its unique nutritional requirements. Different culture conditions may be required to determine if the 4 main BRD bacterial species can all reside within multi-species biofilms.

Studies on the responses of polymicrobial BRD biofilms to antimicrobials are lacking. Polymicrobial biofilms can show higher recalcitrance to antimicrobials through a number of mechanisms including enzymatic inactivation, changes in the expression of antimicrobial resistance genes (ARG), exchange of ARGs among members within the biofilm, inhibition of electron transport or altered membrane fluidity (67). In the case of the *Pasteurellaceae*, horizontal gene transfer is of particular interest due to the presence of integrative and conjugative elements (ICE) and other mobile genetic elements that readily promote the exchange of ARGs among members possibly within a biofilm community. *Pasteurellaceae* ICE can contain up to 12 different ARGs that can be transferred from one bacterial cell to another in a single event (68). A polymicrobial BRD biofilm could represent a scenario where *Pasteurellaceae* species coexist as a community in close proximity, thereby dramatically increasing the opportunity for exchange of ARGs.

## 5 How biofilms affect BRD antimicrobial treatment

The Calgary Biofilm Device (CBD) (69) was used to evaluate the susceptibility of *H. somni*, *M. haemolytica*, *P. multocida*, and *T. pyogenes* (*Arcanobacterium pyogenes* at the time) biofilms to several antimicrobials *in vitro* (36). The isolates tested originated from field cases of cattle with BRD. Interestingly, free-living and sessile *P. multocida* and *H. somni* cells showed similar sensitivities, whereas *M. haemolytica* and *T. pyogenes* biofilms were less sensitive than planktonic cells to 4/7 and 7/7 antimicrobials, respectively. Boukahil and Czuprynski also reported that *M. haemolytica* (a single isolate from a pneumonic bovine lung) biofilms exhibited increased resistance to 5 different antimicrobials (chlortetracycline, erythromycin, gentamycin, tulathromycin, and florfenicol) as compared to planktonic cells (28). The authors also noted that it required higher concentrations of antimicrobials to eradicate biofilms (MBEC) formed on epithelial cells as compared to polystyrene. It is possible that differences in methodology may account for the variability in antimicrobial sensitivity, as the medium used to grow bacteria can interfere with the architectural formation and density of biofilms (4, 29).

Another study investigated the possible relationship between the tilmicosin (TIL) MIC of *M. haemolytica* and *P. multocida* with BRD outcome after TIL treatment (70). The bacterial isolates were obtained from the upper respiratory tract and only those samples that provided either *M. haemolytica* or *P. multocida* were included. Among calves that had *M. haemolytica* or *P. multocida* with a susceptible TIL-MIC, only 61.0 and 63.6% responded to TIL treatment, respectively. Whether biofilms played a role in TIL treatment failure was not investigated.

When a bacterial biofilm is involved in a clinical infection, antimicrobial treatment typically kills planktonic cells, but fails to kill all cells that reside within the biofilm matrix. This may generate a transient relief of symptoms followed by the recurrence of clinical symptoms as new bacterial cells detach from the biofilm and reinitiate infection (22). Most standard antimicrobial susceptibility testing of bacteria is carried out using planktonic cells (71). Some have proposed that pneumonic pasteurellosis responds favorably to antimicrobials when treated at the onset of the disease, whereas pneumonia caused by *H. somni* or *M. bovis* does not (72). Others have reported an overall BRD antimicrobial treatment success rate of 70% (73, 74). Although biofilms may contribute to treatment failure, other factors such as interactions between the antimicrobials administrated, the method of administration, host, and the environment all likely play a role in the success or failure of antimicrobial therapy (73).

Non-attached aggregate biofilms (also known as microcolonies) have been observed in human open wounds, the lungs of cystic fibrosis patients, otitis media infections, and in soft tissue fillers. These aggregates have also been observed *in vitro* in liquid media used to grow *P. aeruginosa* (75). Interestingly, biofilm aggregates can exhibit similar phenotypic characteristics to that of surface-attached biofilms such as higher AMR (75). Susceptibility testing standards recommend to incubate planktonic cultures of *M. haemolytica*, *P. multocida*, *H. somni*, and *T. pyogenes* in microdilution for 18–24 h (71, 76). For *M. bovis* and *B. trehalosi*, there is no current antimicrobial susceptibility test (AST) standards (76), and different incubation times ranging from 48 to 96 h have been reported in the literature for *M. bovis*. Whether BRD bacteria form biofilm aggregates in broth during the aforementioned incubation times for AST is unknown.

## 6 Methodologies employed to study bacteria biofilms

**Figure 3:**
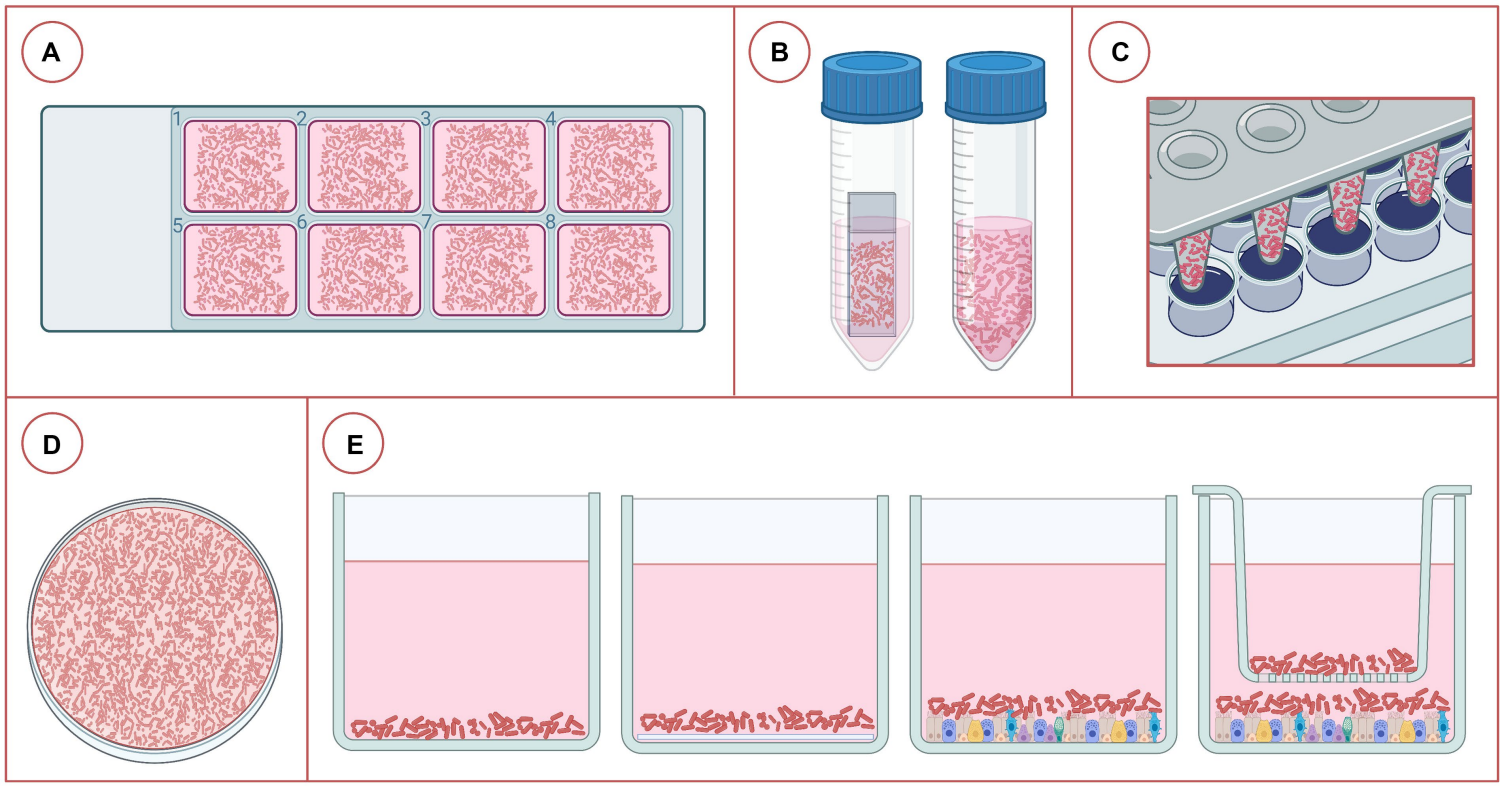
Different platforms used for the study of bacterial biofilms associated with bovine respiratory disease. **(A)** Eight-chamber cover glass slides, **(B)** coverslip in a tube or just the tube with growth medium, **(C)** Calgary Biofilm Device, **(D)** petri dish containing liquid medium, **(E)** plates with 6, 24, 48, or 96 wells, from left to right: just liquid medium, liquid medium + coverslip, medium + fixed eukaryotic cells (2D culture), and medium + fixed eukaryotic cells + transwell (2D culture). *Created in BioRender*.

Different growth media can influence biofilm architecture and its response to antimicrobials (4). For instance, Boukahil et al. reported higher MBECs for biofilms grown in the presence of primary bovine bronchial epithelial cells (BBEC) compared to those grown in 24-well polystyrene plates (28). Incubation time can impact maturity and growth of the biofilm, with differences occurring across bacterial species. Currently, there are numerous protocols that have been employed to assess the ability of BRD bacteria to form biofilms (Supplementary Table 1). This makes it challenging to compare results across studies. However, some common methodology features have been employed (Supplementary Table 1). With *M. haemolytica*, incubation times of at least 36 h are needed to form robust biofilms (24). With a few exceptions, at least 48 h of incubation are needed to form *P. multocida* biofilms. Likewise, *H. somni* biofilms require at least 48 h to form and shaking during incubation should be avoided to minimize aeration (30, 40). Regarding *T. pyogenes*, there seems to be good agreement about the media to use to grow biofilms (BHI supplemented with fetal bovine serum) for at least 24 h. Most of the *in vitro* biofilm studies with BRD bacteria conducted to date have investigated the formation of biofilms on abiotic surfaces like polystyrene plates, glass coverslips or tubes as opposed to eukaryotic cells (Figure 3).

Other less common approaches to studying BRD bacteria biofilms were found in the literature. The Calgary Biofilm Device (CBD) consists of pegs attached to a lid that fits inside a 96-well microplate containing growth medium that can be inoculated with bacteria, enabling high throughput antimicrobial susceptibility testing (AST; Supplementary Table 1; Figure 3). In another study, BBECs were fixed in tissue culture wells to prevent their detachment as result of the accumulation of bacterial end products after 12 h of incubation (28). This method had minimal effect on epithelial cells or tertiary protein structure, making it suitable for biofilm studies. The authors also fixed BBECs in joined wells (66) to investigate the impact that competitive inhibition may have on the formation of biofilms by *M. haemolytica* and *P. multocida* (66). Lastly, polymicrobial biofilms have been used to study *M. haemolytica – P. multocida* and *H. somni – P. multocida* interactions (40, 66), but surprisingly few of these studies have been undertaken even though BRD is clearly a polymicrobial disease.

Aside from the medium and incubation time used to grow biofilms, the presence of mammalian cells to mimic the natural host environment also influences biofilm characteristics. With regard to BRD bacteria, most biofilm studies have grown bacteria in a variety of broth media, and only in a couple of instances have they been formed using a submerged tissue system (STC) (28, 66). However, STCs have limitations in the study of host-pathogen interactions because they lack the diversity of cell types present in host tissues (77).

Air-liquid interface (ALI) systems are an alternative to STCs, but presently they have only be used to elucidate host-pathogen interactions for *M. haemolytica*, not to specifically investigate the impact of biofilms on AMR (66, 77). ALIs allow for cell differentiation and the formation of tight junctions, which are more representative of host tissues, but still have some limitations. For example, they do not fully represent the eukaryotic tissue architecture and the differentiation of eukaryotic cells requires prolonged incubation periods (14–42 d) (77). Ideally, the assembly of 3D cell culture systems or airway organoids, combined with multi-bacterial species approaches could provide new insight into the possible impact of BRD bacteria biofilms on antimicrobial susceptibility. The development of 3D bovine airway organoid models would provide a more accurate representation of the architecture, composition, and environment of host tissues (77).

*In vivo* detection, direct or indirectly, of BRD bacterial biofilms has been reported in the literature (17, 18, 32, 40). Additionally, there is direct evidence supporting the existence of *in vivo P. multocida* biofilms in pulmonary tissue from chickens suffering from induced-chronic avian cholera (19). Different techniques have been employed with different tissues to verify biofilm formation including; histopathology, crystal violet (CV) staining, fluorescein isothiocyanate (FITC)-conjugated *Griffonia simplicifolia* lectin (GS-II), fluorescent *in situ* hybridization (FISH), and transmission (TEM) and scanning electron microscopy (SEM: Supplementary Table 1).

## 7 Treatments targeting BRD bacterial biofilms

As new technologies are developed and improved, so are novel approaches to fight biofilm recalcitrant infections. An example is the use of CRISPR-Cas to prevent the formation of *E. coli* biofilms in urinary catheters (78). In this work, genes related to bacterial cell adhesion, QS, and biofilm formation were targeted by CRISPR-Cas9, resulting in a reduction in biofilm formation. Likewise, the use of regulatory micro RNAs (miRNA) has the potential to enhance the ability of antimicrobials to control biofilms. Human airway epithelial cells (AEC) secret miRNA in extracellular vesicles (EV) that have been shown to decrease *P. aeruginosa* biofilm formation and reduce resistance to beta-lactam antibiotics (79). A comprehensive review of the current strategies that are being explored to combat biofilms is beyond the scope of this article, but Uruen et al. provides an excellent overview. Within the context of AMR, studies addressing anti-biofilm forming strategies for the bacteria involved in BRD are limited, with only three peer-reviewed articles being identified (80–82).

Aside from causing suppurative bronchopneumonia in cattle, *P. multocida* is the causative agent of atrophic rhinitis in swine. Both mycophenolate mofetil and indocyanine green inhibited biofilm formation by *P. multocida* serotypes A and D in swine, but were ineffective against preformed biofilms (80). In this scenario, these molecules could possibly be used to prevent the formation of biofilms (80). However, the authors did not elaborate on the possibility of these compounds adversely effecting animal health or their likelihood of meeting the requirement for regulatory approval. Another study evaluated the biofilm-killing properties of several essential oils on bacteria related to porcine respiratory infections (81). However, *P. multocida* was not included in the experiment based on the authors assumption it was not capable of forming strong biofilms. However, as outlined above, *P. multocida* has been shown to form biofilms *in vitro*. Considering that thyme and winter savory oils disrupted stablished *Streptococcus suis* and *Actinobacillus pleuropneumoniae* biofilms, such additives may have value in *P. multocida* biofilms, but are likely to be more relevant altering intestinal as opposed to respiratory microbiomes. A third study described the biofilm inhibitory properties of two different ethanol extracts which were shown to be effective against *P. multocida* with minimal negative effects on host tissues (82).

Regarding *T. pyogenes*, the Phage vB_EcoM-UFV13 (UFV13 in short) was effective in reducing *T. pyogenes* (isolated from dairy cattle) cell adhesion and decreasing biofilm formation (58). Nanomaterials are emerging as potential antimicrobial agents. In a different study, silver nanoparticles (AgNP) targeting multidrug resistant *T. pyogenes* (dairy cattle clinical endometritis) decreased bacterial cell viability and biofilm formation by increasing oxidative stress (83). More recently, luteolin, a natural flavonoid found in plants, inhibited *T. pyogenes* biofilm-formation *in vitro* and was able to disperse pre-formed biofilms by decreasing TatD DNases binding to extracellular DNA (84). Additionally, luteolin significantly reduced clinical symptoms in a rat endometritis model caused by *T. pyogenes* (60). However, the *in vivo* presence of *T. pyogenes* biofilms was demonstrated on contact lenses pre-introduced in the rat uterus rather than on the uterus tissue itself.

## 8 Conclusion and future directions

The field of BRD bacteria biofilms has significant knowledge gaps. Further research would be of interest for the livestock industry considering their potential to increase AMR in BRD bacteria (9) and to enhance the exchange of ARGs (14, 15). The *in vivo* study of BRD bacterial biofilms in clinically healthy and ill calves would help define their role in pathogenesis (whether during chronic infection, opportunistic state in healthy calves, or both) and possibly shed insight into improved approaches for antimicrobial therapy.

Bacterial aggregate biofilms are either free-floating or embedded in the host tissues and present the traditional (surfaced-attached) biofilm mode of growth (1). Considering that bacteria involved in chronic infections in humans tend to aggregate but not necessarily attach to a surface (75), the possible presence of biofilm aggregates of BRD bacteria in host tissues should be further explored. Particularly when one considers that these could promote the mass movement of BRD bacteria into the lower lung. Filtering and cloning is a common technique used in the isolation and purification of mycoplasmas from biological samples as it is assumed that their cells naturally aggregate and exist as clumps of mixed strains (85, 86). The small cellular diameter and lack of a cell wall of mycoplasmas enables individual, pleomorphic cells to pass through a 0.22 μm filter, whereas cell aggregates are trapped on the filter membrane. Moreover, the presence of mycoplasma cell clumps was demonstrated *in vitro* as a larger bacterial cell count was recovered after liquid growth/ aggregates were subjected to freeze/ thaw cycles or sonication (87). Therefore, the evaluation of whether mycoplasma cell clumps represent non-adherent biofilm aggregates should also be further explored.

Lastly, the existence of BRD bacteria biofilm aggregates in *in vitro* susceptibility testing systems, like broth microdilution should be investigated to determine if they have an impact on ASTs. Higher MBEC were observed when *M. haemolytica* biofilms were grown on epithelial cells as compared to polystyrene (28). This highlights the importance of developing standard AST protocols for BRD bacteria biofilms in order to further assess their possible role in antimicrobial failure and enable comparison of studies across laboratories. For this, a consensus on the definition of a robust and mature biofilm for each BRD bacterial species should be reached to avoid testing antimicrobials on biofilms at different stages of development (75). Aside from viruses, different BRD bacterial species can coexist within healthy or diseased, cattle (14, 15). Following a polymicrobial approach in the study of biofilms (40, 88) would shed light into possible synergisms within the BRD bacterial complex. Coupling 3D tissue systems with multi-omics approaches (i.e., genomics, transcriptomics, metabolomics) would generate insight into the bacterial genes / virulence factors associated with biofilms and the challenge they pose to antimicrobial therapy.

## Author contributions

TM: Project administration, Resources, Supervision, Writing – review & editing. SA-L: Conceptualization, Writing – original draft. RZ: Conceptualization, Writing – review & editing. MJ: Funding acquisition, Resources, Writing – review & editing.

## Footnotes

^1^ https://pubmed.ncbi.nlm.nih.gov/

^2^ https://www.scopus.com/

## References

[1] Sauer K Stoodley P Goeres DM Hall-Stoodley L Burmolle M Stewart PS. The biofilm life cycle: expanding the conceptual model of biofilm formation. Nat Rev Microbiol. (2022) 20:608–20. doi: 10.1038/s41579-022-00767-0, PMID: 35922483 PMC9841534

[2] Nesse LL Osland AM Vestby LK. The role of biofilms in the pathogenesis of animal bacterial infections. Microorganisms. (2023) 11:1–22. doi: 10.3390/microorganisms11030608, PMID: 36985183 PMC10059901

[3] Kragh KN Hutchison JB Melaugh G Rodesney C Roberts AE Irie Y. Role of multicellular aggregates in biofilm formation. MBio. (2016) 7:e00237. doi: 10.1128/mBio.00237-16, PMID: 27006463 PMC4807362

[4] Uruen C Chopo-Escuin G Tommassen J Mainar-Jaime RC Arenas J. Biofilms as promoters of bacterial antibiotic resistance and tolerance. Antibiotics (Basel). (2020) 10:1–36. doi: 10.3390/antibiotics10010003 33374551 PMC7822488

[5] Olivares E Badel-Berchoux S Provot C Prevost G Bernardi T Jehl F. Clinical impact of antibiotics for the treatment of *Pseudomonas aeruginosa* biofilm infections. Front Microbiol. (2019) 10:2894. doi: 10.3389/fmicb.2019.02894 31998248 PMC6962142

[6] Romling U Balsalobre C. Biofilm infections, their resilience to therapy and innovative treatment strategies. J Intern Med. (2012) 272:541–61. doi: 10.1111/joim.12004, PMID: 23025745

[7] Petruzzi B Briggs RE Tatum FM Swords WE De Castro C Molinaro A. Capsular polysaccharide interferes with biofilm formation by *Pasteurella multocida* serogroup a. MBio. (2017) 8:1–17. doi: 10.1128/mBio.01843-17, PMID: 29162713 PMC5698555

[8] Griffin D Chengappa MM Kuszak J McVey DS. Bacterial pathogens of the bovine respiratory disease complex. Vet Clin North Am Food Anim Pract. (2010) 26:381–94. doi: 10.1016/j.cvfa.2010.04.004 20619191

[9] Andres-Lasheras S Jelinski M Zaheer R McAllister TA. Bovine respiratory disease: conventional to culture-independent approaches to studying antimicrobial resistance in North America. Antibiotics (Basel). (2022) 11:1–26. doi: 10.3390/antibiotics11040487 PMC9025279 35453238

[10] Hilton WM. BRD in 2014: where have we been, where are we now, and where do we want to go? Anim Health Res Rev. (2014) 15:120–2. doi: 10.1017/S1466252314000115, PMID: 25358813

[11] Smith RA Step DL Woolums AR. Bovine respiratory disease: looking back and looking forward, what do we see? Vet Clin North Am Food Anim Pract. (2020) 36:239–51. doi: 10.1016/j.cvfa.2020.03.009 32451026

[12] Radostits OM Gay CC Hinchcliff KW Constable PD. Veterinary medicine In: A textbook of the diseases of cattle, horses, sheep, pigs and goats. Eds. Rodenhuis J Demetriou-Swanwick R. 10th Edition Toronto, Canada: Elsevier (2010).

[13] Booker CW Lubbers BV. Bovine respiratory disease treatment failure: impact and potential causes. Vet Clin North Am Food Anim Pract. (2020) 36:487–96. doi: 10.1016/j.cvfa.2020.03.007 32451037

[14] Andres-Lasheras S Ha R Zaheer R Lee C Booker CW Dorin C. Prevalence and risk factors associated with antimicrobial resistance in bacteria related to bovine respiratory disease-a broad cross-sectional study of beef cattle at entry into Canadian feedlots. Front Vet Sci. (2021) 8:692646. doi: 10.3389/fvets.2021.692646 34277758 PMC8280473

[15] Klima CL Zaheer R Cook SR Booker CW Hendrick S Alexander TW. Pathogens of bovine respiratory disease in north American feedlots conferring multidrug resistance via integrative conjugative elements. J Clin Microbiol. (2014) 52:438–48. doi: 10.1128/JCM.02485-13, PMID: 24478472 PMC3911356

[16] Thanner S Drissner D Walsh F. Antimicrobial Resistance in Agriculture. MBio. (2016) 7:e02227–15. doi: 10.1128/mBio.02227-15, PMID: 27094336 PMC4850276

[17] Sandal I Shao JQ Annadata S Apicella MA Boye M Jensen TK. *Histophilus somni* biofilm formation in cardiopulmonary tissue of the bovine host following respiratory challenge. Microbes Infect. (2009) 11:254–63. doi: 10.1016/j.micinf.2008.11.011, PMID: 19095078

[18] Morck DW Olson ME Acres SD Daoust PY Costerton JW. Presence of bacterial glycocalyx and fimbriae on *Pasteurella haemolytica* in feedlot cattle with pneumonic pasteurellosis. Can J Vet Res. (1989) 53:167–71. PMID: 2565756 PMC1255542

[19] Petruzzi B Dalloul RA LeRoith T Evans NP Pierson FW Inzana TJ. Biofilm formation and avian immune response following experimental acute and chronic avian cholera due to *Pasteurella multocida*. Vet Microbiol. (2018) 222:114–23. doi: 10.1016/j.vetmic.2018.07.005, PMID: 30080666

[20] Hermeyer K Jacobsen B Spergser J Rosengarten R Hewicker-Trautwein M. Detection of *Mycoplasma bovis* by in-situ hybridization and expression of inducible nitric oxide synthase, nitrotyrosine and manganese superoxide dismutase in the lungs of experimentally-infected calves. J Comp Pathol. (2011) 145:240–50. doi: 10.1016/j.jcpa.2010.12.005 21334636

[21] McAuliffe L Ellis RJ Miles K Ayling RD Nicholas RAJ. Biofilm formation by mycoplasma species and its role in environmental persistence and survival. Microbiology (Reading). (2006) 152:913–22. doi: 10.1099/mic.0.28604-0, PMID: 16549656

[22] Costerton JW Stewart PS Greenberg EP. Bacterial biofilms: a common cause of persistent infections. Science. (1999) 284:1318–22. doi: 10.1126/science.284.5418.1318 10334980

[23] Subhadra B Cao D Jensen R Caswell C Inzana TJ. Identification and initial characterization of Hfq-associated sRNAs in *Histophilus somni* strain 2336. PLoS One. (2023) 18:e0286158. doi: 10.1371/journal.pone.0286158, PMID: 37220152 PMC10204968

[24] Boukahil I Czuprynski CJ. Characterization of *Mannheimia haemolytica* biofilm formation in vitro. Vet Microbiol. (2015) 175:114–22. doi: 10.1016/j.vetmic.2014.11.012, PMID: 25480166

[25] Haig S-H. Adherence of *Mannheimia haemolytica* to ovine bronchial epithelial cells. Biosci Horiz. (2011) 4:50–60. doi: 10.1093/biohorizons/hzr007

[26] Montes Garcia JF Vaca S Delgado NL Uribe-Garcia A Vazquez C Sanchez Alonso P. *Mannheimia haemolytica* OmpP2-like is an amyloid-like protein, forms filaments, takes part in cell adhesion and is part of biofilms. Antonie Van Leeuwenhoek. (2018) 111:2311–21. doi: 10.1007/s10482-018-1122-9, PMID: 29974354

[27] Figueroa-Valenzuela C Montes-Garcia JF Vazquez-Cruz C Zenteno E Pereyra MA Negrete-Abascal E. *Mannheimia haemolytica* OmpH binds fibrinogen and fibronectin and participates in biofilm formation. Microb Pathog. (2022) 172:105788. doi: 10.1016/j.micpath.2022.105788, PMID: 36126788

[28] Boukahil I Czuprynski CJ. *Mannheimia haemolytica* biofilm formation on bovine respiratory epithelial cells. Vet Microbiol. (2016) 197:129–36. doi: 10.1016/j.vetmic.2016.11.012, PMID: 27938674 PMC7126505

[29] Pillai DK Cha E Mosier D. Role of the stress-associated chemicals norepinephrine, epinephrine and substance P in dispersal of *Mannheimia haemolytica* from biofilms. Vet Microbiol. (2018) 215:11–7. doi: 10.1016/j.vetmic.2017.11.025, PMID: 29426400

[30] Sandal I Hong W Swords WE Inzana TJ. Characterization and comparison of biofilm development by pathogenic and commensal isolates of *Histophilus somni*. J Bacteriol. (2007) 189:8179–85. doi: 10.1128/JB.00479-07, PMID: 17644581 PMC2168709

[31] O'Toole D Sondgeroth KS Somni H In: Aktories K, editor. Biology, molecular basis of pathogenesis, and host immunity. Blacksburg, VA: Springer (2016)

[32] O'Toole D Hunter R Allen T Zekarias B Lehmann J Kim KS. Effect of *Histophilus somni* on heart and brain microvascular endothelial cells. Vet Pathol. (2017) 54:629–39. doi: 10.1177/0300985817691581 28178428

[33] Elswaifi SF Scarratt WK Inzana TJ. The role of lipooligosaccharide phosphorylcholine in colonization and pathogenesis of *Histophilus somni* in cattle. Vet Res. (2012) 43:49. doi: 10.1186/1297-9716-43-49, PMID: 22676226 PMC3406970

[34] Sandal I Inzana TJ Molinaro A De Castro C Shao JQ Apicella MA. Identification, structure, and characterization of an exopolysaccharide produced by *Histophilus somni* during biofilm formation. BMC Microbiol. (2011) 11:186. doi: 10.1186/1471-2180-11-186, PMID: 21854629 PMC3224263

[35] Pan Y Subhadra B Sandal I Dickerman A Inzana TJ. The role of uspE in virulence and biofilm formation by *Histophilus somni*. Vet Microbiol. (2021) 263:109267. doi: 10.1016/j.vetmic.2021.109267, PMID: 34739965

[36] Olson ME Ceri H Morck DW Buret AG Read RR. Biofilm bacteria: formation and comparative susceptibility to antibiotics. Can J Vet Res. (2002) 66:86–92. PMID: 11989739 PMC226988

[37] Saha O Islam MR Rahman MS Hoque MN Hossain MA Sultana M. First report from Bangladesh on genetic diversity of multidrug-resistant *Pasteurella multocida* type B:2 in fowl cholera. Vet World. (2021) 14:2527–42. doi: 10.14202/vetworld.2021.2527-2542, PMID: 34840474 PMC8613801

[38] Prajapati A Chanda MM Dhayalan A Yogisharadhya R Chaudhary JK Mohanty NN. Variability in in vitro biofilm production and antimicrobial sensitivity pattern among *Pasteurella multocida* strains. Biofouling. (2020) 36:938–50. doi: 10.1080/08927014.2020.1833192 33059484

[39] Nguyen PV Le CT Nguyen XH Nguyen TM Nguyen KCT. First study on capsular serotypes and virulence factors of *Pasteurella multocida* isolates from phan rang sheep in Vietnam. Vet World. (2023) 16:281–90. doi: 10.14202/vetworld.2023.281-290, PMID: 37042011 PMC10082718

[40] Petruzzi B Dickerman A Lahmers K Scarratt WK Inzana TJ. Polymicrobial biofilm interaction between Histophilus somni and *Pasteurella multocida*. Front Microbiol. (2020) 11:1561. doi: 10.3389/fmicb.2020.01561, PMID: 32754136 PMC7366659

[41] Kachlany SC Planet PJ Bhattacharjee MK Kollia E DeSalle R Fine DH. Nonspecific adherence by *Actinobacillus actinomycetemcomitans* requires genes widespread in bacteria and archaea. J Bacteriol. (2000) 182:6169–76. doi: 10.1128/JB.182.21.6169-6176.2000, PMID: 11029439 PMC94753

[42] Peng Z Wang X Zhou R Chen H Wilson BA Wu B. *Pasteurella multocida*: genotypes and genomics. Microbiol Mol Biol Rev. (2019) 83:1–37. doi: 10.1128/MMBR.00014-19 PMC6759666 31484691

[43] Zhan L Zhang J Zhao B Li X Zhang X Hu R. Genomic and transcriptomic analysis of bovine *Pasteurella multocida* serogroup a strain reveals insights into virulence attenuation. Front Vet Sci. (2021) 8:765495. doi: 10.3389/fvets.2021.765495, PMID: 34859092 PMC8631534

[44] Prajapati A Yogisharadhya R Mohanty NN Mendem SK Nizamuddin A Chanda MM. Comparative genome analysis of *Pasteurella multocida* serogroup B:2 strains causing haemorrhagic septicaemia (HS) in bovines. Gene. (2022) 826:146452. doi: 10.1016/j.gene.2022.146452, PMID: 35339640

[45] de Emery BD Furian TQ Pilatti RM Chitolina GZ Borges KA Salle CTP. Evaluation of the biofilm formation capacity of *Pasteurella multocida* strains isolated from cases of fowl cholera and swine lungs and its relationship with pathogenicity. Pesqui Vet Bras. (2017) 37:1041–8. doi: 10.1590/s0100-736x2017001000001

[46] Wang Z Li L Liu P Wang C Lu Q Liu L. Role of aspartate ammonia-lyase in *Pasteurella multocida*. BMC Microbiol. (2020) 20:369. doi: 10.1186/s12866-020-02049-2, PMID: 33272193 PMC7713322

[47] Yang Y Hu P Gao L Yuan X Hardwidge PR Li T. Deleting qseC downregulates virulence and promotes cross-protection in *Pasteurella multocida*. Vet Res. (2021) 52:140. doi: 10.1186/s13567-021-01009-6, PMID: 34801081 PMC8605557

[48] Xu T Zheng Y Liu B Kou M Jiang Q Liu J. Pmorf0222, a virulence factor in *Pasteurella multocida*, activates nuclear factor kappa B and mitogen-activated protein kinase via toll-like receptor 1/2. Infect Immun. (2023) 91:e0019322. doi: 10.1128/iai.00193-22, PMID: 36541752 PMC9872710

[49] Calcutt MJ Lysnyansky I Sachse K Fox LK Nicholas RAJ Ayling RD. Gap analysis of *Mycoplasma bovis* disease, diagnosis and control: an aid to identify future development requirements. Transbound Emerg Dis. (2018) 65:91–109. doi: 10.1111/tbed.12860, PMID: 29582590

[50] Sirand-Pugnet P Citti C Barre A Blanchard A. Evolution of mollicutes: down a bumpy road with twists and turns. Res Microbiol. (2007) 158:754–66. doi: 10.1016/j.resmic.2007.09.007, PMID: 18023150

[51] Citti C Dordet-Frisoni E Nouvel LX Kuo CH Baranowski E. Horizontal gene transfers in mycoplasmas (Mollicutes). Curr Issues Mol Biol. (2018) 29:3–22. doi: 10.21775/cimb.029.003, PMID: 29648541

[52] Nicholas R Ayling RD McAuliffe L. Mycoplasma diseases of ruminants. Wallingford, Oxfordshire: CABI (2008).

[53] Gonzalez RN Wilson DJ. Mycoplasmal mastitis in dairy herds. Vet Clin North Am Food Anim Pract. (2003) 19:199–221. doi: 10.1016/S0749-0720(02)00076-2 12682943

[54] Justice-Allen A Trujillo J Corbett R Harding R Goodell G Wilson D. Survival and replication of Mycoplasma species in recycled bedding sand and association with mastitis on dairy farms in Utah. J Dairy Sci. (2010) 93:192–202. doi: 10.3168/jds.2009-2474, PMID: 20059918

[55] Soehnlen MK Aydin A Murthy KS Lengerich EJ Hattel AL Houser BA. Epidemiology of *Mycoplasma bovis* in Pennsylvania veal calves. J Dairy Sci. (2012) 95:247–54. doi: 10.3168/jds.2011-4309, PMID: 22192204

[56] Piccinini R Gosney F Snel GGM Luini MV Nicholas RAJ. Environmental survival of *Mycoplasma bovis* on a white veal farm. Vet Rec Case Rep. (2015) 3:1–3. doi: 10.1136/vetreccr-2015-000207

[57] Chen S Hao H Zhao P Ji W Li M Liu Y. Differential immunoreactivity to bovine convalescent serum between *Mycoplasma bovis* biofilms and planktonic cells revealed by comparative immunoproteomic analysis. Front Microbiol. (2018) 9:379. doi: 10.3389/fmicb.2018.00379, PMID: 29556225 PMC5844979

[58] da Silva DV Dias RS Kropinski AM da Silva XA Ferro CG Vidigal PMP. A T4virus prevents biofilm formation by *Trueperella pyogenes*. Vet Microbiol. (2018) 218:45–51. doi: 10.1016/j.vetmic.2018.03.025 29685220

[59] Jost BH Billington SJ. *Arcanobacterium pyogenes*: molecular pathogenesis of an animal opportunist. Antonie Van Leeuwenhoek. (2005) 88:87–102. doi: 10.1007/s10482-005-2316-5, PMID: 16096685

[60] Zhang L Cai Y Li L Chen C Zhao H Zhang Z. Effects of luteolin on biofilm of Trueperella pyogenes and its therapeutic effect on rat endometritis. Int J Mol Sci. (2022) 23:1–14. doi: 10.3390/ijms232214451 PMC9692790 36430929

[61] Zhao K Tian Y Yue B Wang H Zhang X. Virulence determinants and biofilm production among *Trueperella pyogenes* recovered from abscesses of captive forest musk deer. Arch Microbiol. (2013) 195:203–9. doi: 10.1007/s00203-013-0869-7, PMID: 23354327

[62] Ozturk D Turutoglu H Pehlivanoglu F Guler L. Virulence genes, biofilm production and antibiotic susceptibility in *Trueperella pyogenes* isolated from cattle. Israel J Vet Med. (2016) 71:36–42.

[63] Zhao K Li W Huang T Song X Zhang X Yue B. Comparative transcriptome analysis of *Trueperella pyogenes* reveals a novel antimicrobial strategy. Arch Microbiol. (2017) 199:649–55. doi: 10.1007/s00203-017-1338-5, PMID: 28144921

[64] Zhang Z Liang Y Yu L Chen M Guo Y Kang Z. TatD DNases contribute to biofilm formation and virulence in *Trueperella pyogenes*. Front Microbiol. (2021) 12:758465. doi: 10.3389/fmicb.2021.758465, PMID: 34867886 PMC8634637

[65] Rzewuska M Kwiecien E Chrobak-Chmiel D Kizerwetter-Swida M Stefanska I Gierynska M. Pathogenicity and virulence of *Trueperella pyogenes*: a review. Int J Mol Sci. (2019) 20:1–33. doi: 10.3390/ijms20112737, PMID: 31167367 PMC6600626

[66] Boukahil I Czuprynski CJ. Mutual antagonism between Mannheimia haemolytica and *Pasteurella multocida* when forming a biofilm on bovine bronchial epithelial cells in vitro. Vet Microbiol. (2018) 216:218–22. doi: 10.1016/j.vetmic.2018.02.015, PMID: 29519520

[67] Orazi G O'Toole GA. "it takes a village": mechanisms underlying antimicrobial recalcitrance of polymicrobial biofilms. J Bacteriol. (2019) 202:1–18. doi: 10.1128/JB.00530-19, PMID: 31548277 PMC6932244

[68] Ha R Zaheer R Sargeant C Klima C TA MA. Intra- and inter-species horizontal transfer potential of integrative and conjugative elements carrying antimicrobial resistance genes. Sherbrooke, QC, Canada: Canadian Society of Microbiologists (2019).

[69] Ceri H Olson ME Stremick C Read RR Morck D Buret A. The Calgary biofilm device: new technology for rapid determination of antibiotic susceptibilities of bacterial biofilms. J Clin Microbiol. (1999) 37:1771–6. doi: 10.1128/JCM.37.6.1771-1776.1999, PMID: 10325322 PMC84946

[70] McClary DG Loneragan GH Shryock TR Carter BL Guthrie CA Corbin MJ. Relationship of in vitro minimum inhibitory concentrations of tilmicosin against Mannheimia haemolytica and Pasteurella multocida and in vivo tilmicosin treatment outcome among calves with signs of bovine respiratory disease. J Am Vet Med Assoc. (2011) 239:129–35. doi: 10.2460/javma.239.1.129, PMID: 21718206

[71] CLSI. Performance standards for antimicrobial disk and dilution susceptibility tests for bacteria isolated from animals. CLSI standard VET01. Eds. Leirer KL Jenkins AEM Martin L. 5th Wayne, PA: Clinical and Laboratory Standards Institute (2018)

[72] Constable PD Hinchcliff KW Done SH Grünberg W. Veterinary medicine. A textbook of the diseases of cattle, horses, sheep, pigs and goats. Eds. Constable P. D. Hinchcliff K. W. Done S. H. Grünberg W.. 11th Edition St. Louis, Missouri: Elsevier (2017).

[73] Lubbers BV. Pharmacological considerations of antibiotic failures in bovine respiratory disease cases. Anim Health Res Rev. (2020) 21:177–8. doi: 10.1017/S1466252320000122, PMID: 33261709

[74] Avra TD Abell KM Shane DD Theurer ME Larson RL White BJ. A retrospective analysis of risk factors associated with bovine respiratory disease treatment failure in feedlot cattle. J Anim Sci. (2017) 95:1521–7. doi: 10.2527/jas2016.1254 28464093

[75] Alhede M Kragh KN Qvortrup K Allesen-Holm M van Gennip M Christensen LD. Phenotypes of non-attached *Pseudomonas aeruginosa* aggregates resemble surface attached biofilm. PLoS One. (2011) 6:e27943. doi: 10.1371/journal.pone.0027943, PMID: 22132176 PMC3221681

[76] CLSI. Methods for antimicrobial susceptibility testing of infrequently isolated or fastidious bacteria isolated from animals. CLSI standard VET06. Eds. Christopher JP Maritn L Russell MA. 1st ed. Wayne, PA: Clinical and Laboratory Standards Institute (2016)

[77] Gandhi NN Inzana TJ Rajagopalan P. Bovine airway models: approaches for investigating bovine respiratory disease. ACS Infect Dis. (2023) 9:1168–79. doi: 10.1021/acsinfecdis.2c00618, PMID: 37257116

[78] Alshammari M Ahmad A AlKhulaifi M Al Farraj D Alsudir S Alarawi M. Reduction of biofilm formation of *Escherichia coli* by targeting quorum sensing and adhesion genes using the CRISPR/Cas9-HDR approach, and its clinical application on urinary catheter. J Infect Public Health. (2023) 16:1174–83. doi: 10.1016/j.jiph.2023.05.026, PMID: 37271098

[79] Koeppen K Nymon A Barnaby R Bashor L Li Z Hampton TH. Let-7b-5p in vesicles secreted by human airway cells reduces biofilm formation and increases antibiotic sensitivity of *P. aeruginosa*. Proc Natl Acad Sci USA. (2021) 118:1–10. doi: 10.1073/pnas.2105370118 PMC8285967 34260396

[80] Kubera A Thamchaipenet A Shoham M. Biofilm inhibitors targeting the outer membrane protein a of *Pasteurella multocida* in swine. Biofouling. (2017) 33:14–23. doi: 10.1080/08927014.2016.1259415, PMID: 27892689

[81] LeBel G Vaillancourt K Bercier P Grenier D. Antibacterial activity against porcine respiratory bacterial pathogens and in vitro biocompatibility of essential oils. Arch Microbiol. (2019) 201:833–40. doi: 10.1007/s00203-019-01655-7, PMID: 30955056

[82] Qasim N Shahid M Yousaf F Riaz M Anjum F Faryad MA. Therapeutic potential of selected varieties of *Phoenix dactylifera* L. against microbial biofilm and free radical damage to DNA. Dose-Response. (2020) 18:155932582096260. doi: 10.1177/1559325820962609 PMC7573743 33117092

[83] Gurunathan S Choi YJ Kim JH. Antibacterial efficacy of silver nanoparticles on endometritis caused by Prevotella melaninogenica and Arcanobacterum pyogenes in dairy cattle. Int J Mol Sci. (2018) 19:1–20. doi: 10.3390/ijms19041210, PMID: 29659523 PMC5979543

[84] Zhang Z Guo Y Guo Y Zhang L Niu S Tian C. Molecular basis for luteolin as a natural TatD DNase inhibitor in *Trueperella pyogenes*. Int J Mol Sci. (2022) 23:1–13. doi: 10.3390/ijms23158374 PMC9369154 35955509

[85] Tully JG. Cloning and filtration techniques for mycoplasmas Elsevier (1983). 173–177.

[86] Sagne E Citti C Dordet-Frisoni E. Bacterial conjugation protocol for ruminant mycoplasmas. Bio Protoc. (2021) 11:e3893. doi: 10.21769/BioProtoc.3893, PMID: 33732782 PMC7953245

[87] Dabrazhynetskaya A Furtak V Volokhov D Beck B Chizhikov V. Preparation of reference stocks suitable for evaluation of alternative NAT-based mycoplasma detection methods. J Appl Microbiol. (2014) 116:100–8. doi: 10.1111/jam.12352, PMID: 24112653

[88] Jacques M Malouin F. One health-one biofilm. Vet Res. (2022) 53:51. doi: 10.1186/s13567-022-01067-4, PMID: 35799278 PMC9264708

